# Evaluation of a preservative-free, cross-linked sodium hyaluronate-based solution, Lacri +^®^, in dogs with dry eye: a pilot trial

**DOI:** 10.1186/s13620-023-00259-4

**Published:** 2023-10-06

**Authors:** Carole Gard, Charles Cassagnes, Sarah Muller, Christelle Navarro, Bruno Jahier

**Affiliations:** 1 MP Labo, 45 Boulevard Marcel Pagnol, 06130 Grasse, France; 2 Clinique VetRiviera, 448 Boulevard du Mercantour, 06200 Nice, France

**Keywords:** Dry eye, Dog, Tear supplement, Lubricant, Lacrimimetic, Eyedrops, Preservative-free

## Abstract

**Background:**

The purpose of this pilot trial was to evaluate the efficacy and benefits of a preservative-free cross-linked sodium hyaluronate solution (Lacri +^®^, MP Labo, France) in 19 privately-owned dogs with dry eye. The animals were administered 2 drops of the tested product in each affected eye, twice a day (BID) for 30 days. Improvement in the global ocular clinical score (sum of the individual scores for conjunctivitis, ocular discharge, eye irritation, and corneal opacity/pigmentation/vascularization, each rated from 0 to 3) was defined as the primary outcome. Besides an improvement in each individual ocular score, tear film quality (Tear Break Up Time, TBUT), dogs’ and owners’ quality of life (QoL), as well as an increase in tear production (Schirmer Tear Test-1, STT1), were considered secondary outcomes. These criteria were assessed on D0, D0 + 15 days, and D0 + 30 days. Finally, a qualitative evaluation of clinical improvement was requested from the owners on D0 + 2, + 15 & + 30 days and from the investigators during the follow-up.

**Results:**

The global clinical ocular score as well as the individual conjunctival and irritation scores improved significantly (*p* < 0.0001) during the pilot trial. The average reduction of the global score reached 30% on D0 + 15 days and 55% on D0 + 30 days compared to D0. Ocular discharge was significantly lower (*p* = 0.0002) on D0 + 30 days compared to baseline; however corneal opacity did not show any significant changes from D0 to the end of the follow-up period. The quantitative tear production was increased at D + 30 (*p* < 0.0001), with a significant improvement as soon as 2 weeks in, with around 30% and 60% of dogs presenting an STT1 value above 10 on D0 + 15 days and on D0 + 30 days, respectively. The QoL score was significantly improved compared to D0 at all time points (*p* < 0.0001). After 2 days of treatment, 39% of the owners rated the efficacy as “good”. The efficacy of the tested product was considered “Good” or “Very Good” by the investigators in 78% and 93% of the cases, on D0 + 15 days and D0 + 30 days, respectively. The tolerance of this preservative-free formulation was good, with only rare and transient minor local reactions, realated to administration rather than the product itself.

## Background

Dry eye (DE) corresponds to a condition of the ocular surface related to a reduction in the aqueous component of the tear film [1]. It is defined as a Schirmer tear test (STT1) reading of less than 15 mm/min, associated to signs such as keratitis, conjunctivitis, ocular discharge, corneal ulceration, or pigmentation in the worse situations. The tear film is commonly characterized as being composed of 3 layers, including a mucus layer close to the corneal epithelial surface, an aqueous layer, and, finally, the most superficial layer of meibomian lipid material that helps to limit evaporation [1]. It is now accepted that this simple distinction is not totally accurate. For instance, the aqueous layer is not only composed of water but also includes cleaved membrane-bound mucins, small soluble mucins, and much larger gel-forming mucins [1]. Therefore, it seems quite challenging to design an adapted tear supplement with rheological properties that could make it a substitute for this complex composition. One cause of dry eye is Keratoconjunctivitis sicca (KCS). The prevalence of KCS in dogs is between 1 and 4% of the population [2], but in predisposed breeds such as English Bulldogs, West Highland White Terriers, Cavalier King Charles Spaniels, American and English Cocker Spaniels or Pugs, it could be as high as 20% [3]. Dry eye can be caused either by a tear deficiency or by an abnormal evaporation due to incomplete eyelid closure [1]. Concurrent systemic diseases such as canine distemper, systemic lupus erythematosus, diabetes mellitus or hypothyroidism can be associated with tear insufficiency, but most of the time, dry eye remains the only diagnosed condition [1]. The etiopathogenesis of tear deficiency can be congenital, infectious, iatrogenic, neurogenic, or immune-mediated. Nevertheless, the individual clinical response can vary and, in many cases, tear substitute eyedrops are still required to provide moisture and lubrication [2, 3]. Several lubricating agents, composed of polyvinyl alcohol, hydroxypropyl methylcellulose, carboxymethylcellulose, polyethylene glycol, or sodium hyaluronate (SH), are currently available. For most of them, multiple instillations per day are recommended. Knowing this therapy will be prescribed to the dog throughout its life, longer-lasting options are required to ease treatment compliance. Sodium hyaluronate is one of the main components of the extracellular matrix. Inside the eye, this polysaccharide (glycosaminoglycan) is naturally found in the aqueous humour and vitreous body and covers the corneal endothelium [3]. In tear supplements, the viscoelasticity of SH made this molecule particularly attractive in limiting tear removal and reinforcing tear stability [3]. The viscoelasticity of SH can vary according to its molecular weight, its concentration, and the presence of ions in the formulation. An additional option to extend the corneal contact time relies on covalently crosslinking the SH. Crosslinking with a molecule with a sustained (adhesive, long lasting) effect and water retention such as urea could be of interest. Moreover, it has been demonstrated that preservatives are part of the vicious circle that characterise dry eye. This trial aims to demonstrate the benefits of a preservative-free, crossed-linked SH-based tear supplement, in dogs suffering from KCS, after 30 days of single use.

## Materials and methods

### Treatment

The tested item consisted in a 4 mg/ml cross-linked sodium hyaluronate solution, presented in a 10 ml preservative-free multidose bottle (Lacri +^®^, MP Labo, Grasse, France).

Owners were instructed to thoroughly clean the eyes of the dogs with a compress soaked with physiological serum prior each test product administration. Two drops of the hyaluronate solution were then instilled into the eye, twice a day (BID) for 30 days. The test product had to be administered at least two hours prior to visiting the investigation site.

### Study design

This multicentric open-label trial was conducted in 4 French veterinary clinics. 5 investigators with referral experience in veterinary ophthalmology (Post-Graduate Certificate in Ophthalmology) took part in the pilot trial.

The study was conducted in accordance with the guidelines on “Good Clinical Practice” (VICH Topic GL9 (GCP), CVMP/VICH/595/98, July 4, 2000) and the guidelines on “Statistical Principles for Veterinary Clinical Trials” (EMEA/CVMP/816/00-FINAL, January 23, 2012). In accordance with the good clinical practice guidelines, this trial was “[…] not likely to cause pain, suffering, distress or lasting harm equivalent to, or higher than, that caused by the introduction of a needle”, and was therefore not subject to Directive 2010/63/EU of the European Parliament and of the Council. In this pilot trial, where all the subjects received the same treatment, owners had to give their written informed consent prior to any enrolment of their dog.

### Animals

To be included, dogs had to show one or several clinical signs suggestive of keratoconjunctivitis sicca such as keratitis, conjunctivitis, chemosis or ocular discharge. Moreover, only dogs with a Schirmer tear test “STT1” with a value between 5 and 10 mm/min were enrolled in the pilot trial.

Dogs were not included if they had either a purulent or mucopurulent ocular discharge, other ocular diseases including corneal ulceration (with a positive fluoresceine test), systemic diseases which could impact ocular clinical signs, or a chronic disease where treatment was known to influence tear secretion. Finally, a wash-out period of 2 days and 2 weeks before inclusion was decided regarding administration of lubricating eyedrops and topical non-steroidal or steroidal anti-inflammatory drugs (NSAIDs or SAIDs), respectively.

### Clinical evaluation

At each visit, dogs underwent a full clinical and ophthalmic examination, using direct and indirect ophthalmoscopy and slit lamp biomicroscopy on D0, D0 + 15 days and D0 + 30 days. Clinical ocular signs corresponding to “conjunctivitis”, “ocular discharge”, “eye irritation”, “corneal opacity/ pigmentation/ vessels” were rated from 0 to 3 based on their intensity). The aggregate score, ranging from 0 to 12, defined as the global ocular clinical score, was the main evaluation criteria of the pilot trial (Table 1). The improvement versus D0 of the mean of each individual ocular score (0–3) was considered to be a secondary outcome.

**Table 1:** Primary outcome: global ocular clinical score. Each clinical sign (conjunctivitis, ocular discharge, eye irritation, corneal opacity/pigmentation/ vessels) is rated from 0 to 3. The global ocular clinical score corresponds to the sum of these individual scores. (Adapted from Williams et al. [2] and Da Silva et al. [4])

Ocular clinical sign	Score
Conjunctivitis	0 = None 1 = Mild hyperaemia 2 = Moderate to severe hyperaemia 3 = Moderate to severe hyperaemia with chemosis
Ocular discharge	0 = None 1 = Mild ocular discharge 2 = Moderate mucoid ocular discharge 3 = Severe mucoid ocular discharge
Eye irritation (frequency of eye blinking and palpebral opening)	0 = None 1 = Some eye blinking 2 = Frequent eye blinking and narrow palpebral opening 3 = Eye is mainly closed
Corneal opacity, pigmentation, and vessels	0 = None 1 = < 25% of the surface 2 = 25% to 50% of the surface 3 = > 50% of the surface

A Schirmer tear test (STT1), a fluorescein test (FT), a tear film break-up time (TBUT), and an intraocular pressure measurement were performed at each visit. The TBUT consists in placing a 1% fluorescein eye drop in the lower fornix, then using a slit-lamp with a bright light setting and a cobalt blue filter to measure the time between the last blink and the first appearance of a dark spot on the cornea (appearance of a dry area). TBUT values of < 10 s are in favour of a tear quality defect.

Qualitative evaluation of clinical improvement was rated as “very good”, “good”, “partial” or “absent” on D0 + 2 days by the owners (scheduled phone call) and as “poor”, “good”, “very good” or “excellent” on D0 + 15 days and on D0 + 30 days by the investigators. Moreover, quality of life (QoL) was assessed by the owners at each time point by completing a questionnaire consisting of 11 questions (based on the “dry eye questionnaire” (5,6) from Caffery (7)). There were four possible answers to each question: not at all (score 0), a little (score 1), quite a bit (score 2), and very much (score 3). Globally, the higher the score, the worse the owner’s and the dog’s condition. The evolution of the mean STT1 (expressed in mm/min), mean TBUT (expressed in seconds) and mean QoL score (0–33) over time were also defined as secondary outcomes.

### Safety evaluation

Tolerance was assessed by both investigators and owners on D0, D0 + 15 days, and D0 + 30 days. All adverse events were recorded.

### Statistical analysis

Data were analysed using the Statgraphics^®^ Centurion XVI software.

The experimental unit was the eye. In case of bilateral KCS, only the right eye was arbitrarily considered for statistical analysis. The significance level for all tests was set at 5%. The mean global ocular clinical score, mean individual ocular scores, mean STT1, mean TBUT, and mean QoL score were compared between D0, D0 + 15 days and D0 + 30 days using a generalized linear mixed effects model (with “time” as a fixed effect and “animal” as a random effect). When significance was reached, pairwise comparisons were performed using the post-hoc Fisher’s least significant difference (LSD) procedure. Moreover, a McNemar’s test was used to compare between time points the percentages of animals in each category regarding qualitative tear production and lacrimal film quality.

## Results

### Characteristics of the study population

Nineteen dogs were enrolled in the pilot trial. Among them, 3 were excluded during the study because of a lack of efficacy of the tested item resulting in a worsening of the ocular condition, and another one for a major deviation. Using the LOCF (Last Observation Carried Forward) principle, the test product efficacy was ultimately assessed in 18 dogs and its safety in 19 dogs.

Eleven (11) breeds were identified in the trial, including Yorkshire Terriers (28%), French Bulldogs, Cockers, Shi-tzus (11% for each), and other breeds (6%). Thirty-three (33) percent of the enrolled dogs were females and 67% were males. One out of two dogs was neutered. The mean age was 7.4 (± 2.8) years with a minimum of 4 years and a maximum of 13 years. The mean weight was 11.1 (± 9.1) kg with a minimum of 3.7 kg and maximum of 35.5 kg.

The clinical signs of dry eyes appeared between 5 and 1153 days (mean: 161 days) prior the inclusion into the study.

### Clinical evaluation

**Fig. 1:**
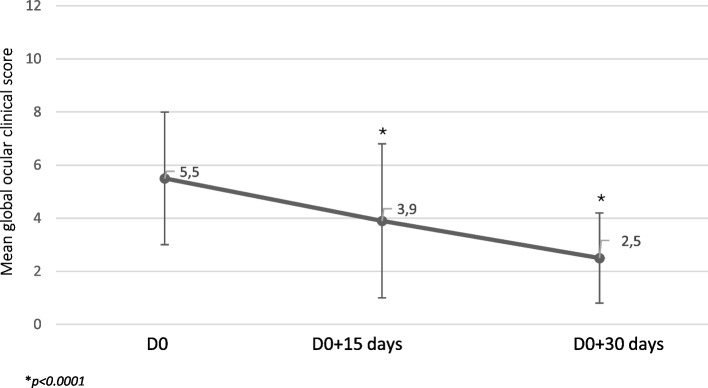
Evolution of the mean global ocular clinical score during the study**.** Clinical ocular signs corresponding to “conjunctivitis”, “ocular discharge”, “eye irritation”, “corneal opacity/ pigmentation/ vessels” were rated from 0 to 3 based on their intensity (Table1). The global ocular clinical score corresponds to the sum of these individual scores

The global clinical score significantly decreased over time with a significant improvement as soon as two weeks after treatment initiation (Fig. 1) (*p* < 0.0001).

The composite score decreased by 31.1% as soon as 2 weeks in. It was more than two times lower (54.5%) at the end of the trial, compared to D0.

**Fig. 2:**
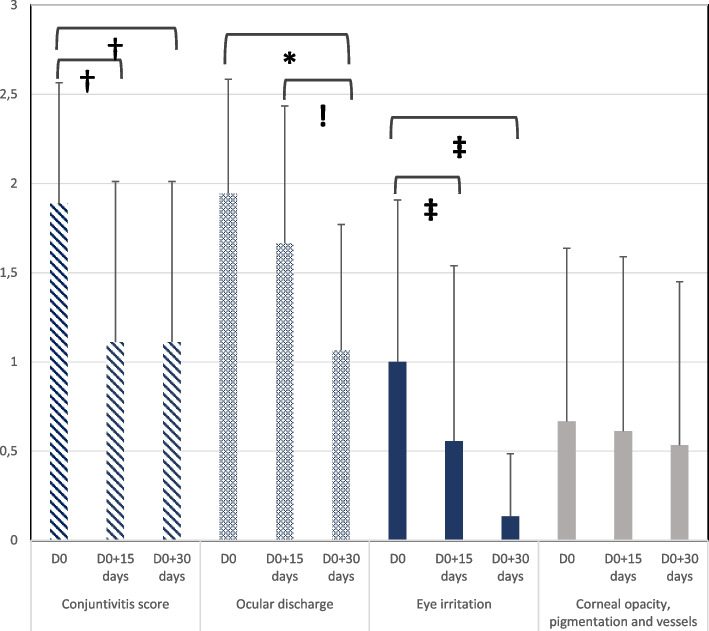
Secondary outcomes: evolution of the mean of individual ocular clinical scores over time. †, ‡ *p* < 0.0001 significant evolution on D0 + 15 days and D0 + 30 days versus D0. * *p* = 0.0002, significant evolution on D0 + 30 days versus D0 and ! *p* = 0.0002, significant evolution on D0 + 30 days versus D0 + 15 days. Individual assessment (conjunctivitis, ocular discharge, eye irritation, corneal opacity/pigmentation/ vessels) were rated from 0 (none) to 3

The mean conjunctivitis and ocular irritation scores steadily decreased over time and were significantly lower compared to D0, as soon as the second visit (*p* < 0.0001, Fig. 2). The mean ocular discharge score regularly decreased over time. Significance was reached at the end of the trial (*p* = 0.0002, Fig. 2). While the study, a slight decrease of the corneal score was noticed. Although no significance was found, this trend was noticeable.

**Fig. 3:**
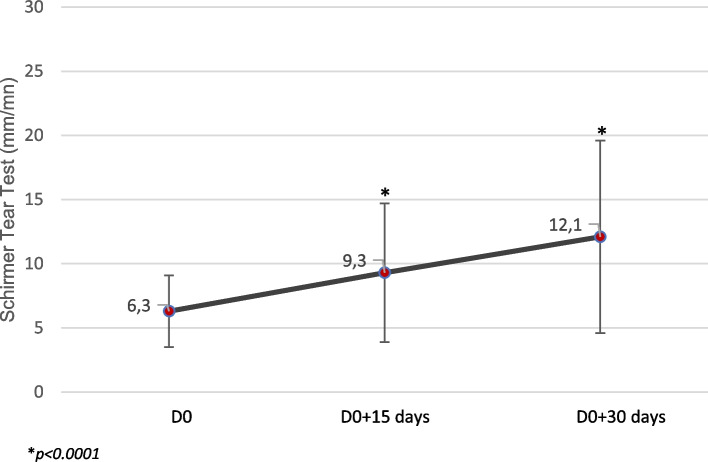
Evolution of tear secretion (Schirmer Tear Test-1) during the study

The Schirmer tear test values increased over the study period. The value of the STT1 (Fig. 3) was found to be significantly different from D0 as soon as 2 weeks in (*p* < 0.0001).

**Fig. 4:**
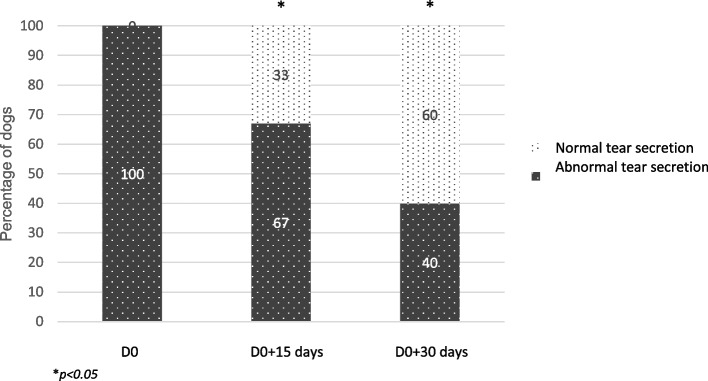
Assessment of tear secretion during the study. Percentage of dogs with STT-1 below or above 10 mm/min

At the end of the pilot trial, 60% of the dogs presented STT1 values above 10 mm/min (Fig. 4). As soon as two weeks after treatment initiation, the proportion of dogs with “normal” STT1 values was significantly higher than at D0 (*p* < 0.05).

The mean TBUT score did not significantly differ over time with 10.1 ± 4.8 s, 10.1 ± 4.2 s and 9.73 ± 4.6 s on D0, D0 + 15 days and D0 + 30 days, respectively. In the same way, the proportion of dogs with “Normal” vs “Abnormal” tear film quality did not significantly increase over time.

**Fig. 5:**
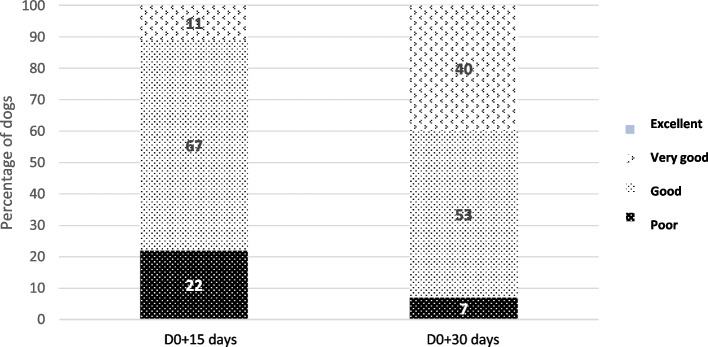
Overall investigators appreciation of product efficacy during the study. Qualitative evaluation of clinical improvement was rated as “poor”, “good”, “very good” or “excellent” on D0 + 15 days and on D0 + 30 days by the investigators

After two days of use, the owners rated the global clinical improvement as “good” in 39% of the cases, “partial” in 33% of the cases, and “absent” in 28% of them. As soon as 2 weeks after the beginning of the treatment, 78% of the investigators rated the improvement of the dogs’ condition “Good” or “Very Good”. This percentage reached 93% at the end of the trial (Fig. 5).

At the end of the trial, 83.3% of the owners considered the device easy to very easy to use. However, 40% of them said that using the bottle became more difficult over time. This can be explained by the conception of the device, as more pressure must be applied when the device is empty (it is part of the manufacturer warnings in the leaflet).

**Fig. 6:**
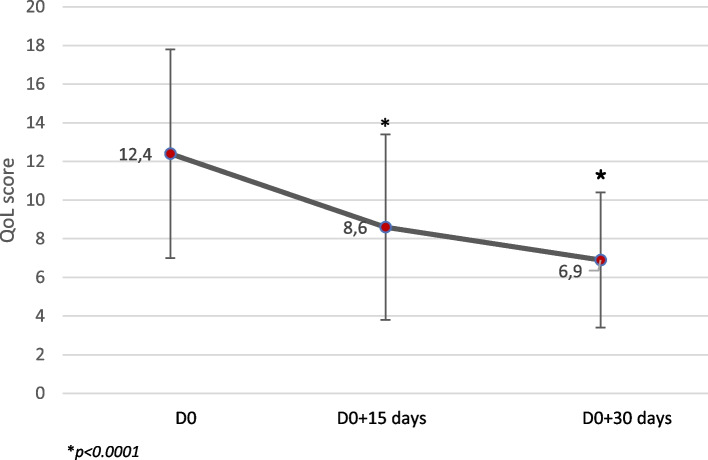
Evolution of the Quality of Life (QoL) score during the study. (*p < 0.0001, significant difference versus D0). QoL was assessed by the owners at each time point by completing a questionnaire consisting of 11 questions (based on the “dry eye questionnaire” [5, 6] from Caffery [7]). For each question four answers were possible: not at all (score 0), a little (score 1), quite a bit (score 2), and very much (score 3)

As soon as two weeks after treatment start and until the end of the study period, the QoL score decreased compared to D0 (*p* < 0.0001), Fig. 6.

The mean percentage of improvement of the QoL score versus D0 reached 23.9% on D0 + 15 days and 38.9% on D0 + 30 days. At the end of the trial, owners gave an average satisfaction rating of 6.1/10.

During the study, fluorescein stain tests were all negative for all dogs, confirming the absence of corneal ulceration. In the same way, the mean intraocular pressure stayed within normal ranges (10-20 mmHg), with no noticeable variations: 16.6 (± 3.6) mmHg on D0, 16.8 (± 3.7) mmHg on D0 + 15 days and 16.7 (± 3.3) mmHg on D0 + 30 days.

### Safety evaluation

The general condition of the 19 dogs included in the safety analysis was rated “good” throughout the trial. None of the investigators or owners reported any side effects. According to the owners, 4 dogs presented minor local reactions. These reactions corresponded either to a slight disturbance in the dog which occasionally kept its eyes closed, blinked after administration, or strongly reacted (2 dogs), or to a possible redness of the eye a few hours after administration (2 dogs). As those reactions rapidly ceased, and as they were only occasionally observed during the trial, they were not considered to be related to the treatment, but rather to the dog’s condition.

## Discussion

In our study, the changes in the ocular clinical signs were consistent with those of previous trials performed with similar products.

Williams et al. [2, 3] have demonstrated the potential benefits of cross-linked SH-based eyedrops in dogs suffering from KCS. The first study was led with no control group and a BID administration of the cross-linked SH gel (3.77 ± 0.09 mg/ml) for 2 weeks [3]. When compared to the previous use of simple SH-based eyedrops, the author concluded that the evolution of conjunctival hyperaemia, ocular irritation, and ocular discharge were significantly better for the cross-linked SH gel (respectively before/after for right eyes: 1.70/0.28, 1.60/0.16, 0.76/0.08). Similarly, in a second controlled, blinded study, where both commercialized non-cross-linked SH and cross-linked SH (3.77 ± 0.09 mg/ml) gels were administered three times a day (TID) for 3 weeks [2], conjunctival hyperaemia and irritation intensity exhibited a significantly greater improvement in the cross-linked SH group (respectively before/after for right eyes: 2.2/0.3, 1.5/0.3). Nevertheless, the amount of ocular discharge seemed to be comparable in both groups (before/after for right eyes in the cross-linked SH group: 1.4/0.4) and the author considered this was likely because it was much more variable. Though there was no control group in our pilot trial, a similar trend was observed for ocular discharge, which was only significantly improved at the end of the trial, after one month of use (DO/D30 = 1.94/1.07), whereas conjunctivitis and eye irritation were both significantly improved (*p* < 0,001) as soon as two weeks in (respectively on D15: 1.88/1.11, 1.00/0.56). Despite a constant decrease, the corneal score was not significantly different compared to D0. This finding was not surprising since pigmentation and vessel development on the corneal surface. are persistent clinical signs which often require immunomodulatory drugs. Therefore, many trials testing eye lubricants do not include corneal-related clinical signs [2, 3]. In our trial, we considered it was relevant to include them to evaluate to what extent the product was able to address the whole disease. However, we chose to keep them grouped as a single item (opacity, pigmentation, and vessels) to balance the global score [7, 8].

In our study, the quality of life of both owners and dogs were assessed at each time point in the follow-up period. At the end of the trial, the decrease of the QoL score reached almost 40% compared to D0. The primary aim of the questionnaire was to evaluate how severe the ocular disease was, based on owner perception. As expected, the QoL steadily decreased as clinical signs improved.

Another interesting finding consisted in a significant and persistent increase in tear secretion, even though the product tested was not expected to exhibit any lachrymogenic property. This effect was not reported, for instance, by Williams et al., who were not able to demonstrate a significant increase of the STT values after treatment [2, 3]. Nevertheless, trials performed in a mouse model exposed to “environmental dry eye stress” and treated either with high or low molecular weight SH showed that tear secretion volume significantly decreased with the low-molecular weight SH whereas it remained stable with the high-molecular weight one, which suggests the latter has a protective effect [9]. One limitation of the trial is the possible influence of the product application on the STT values. In a recent article [10], published after the completion of our trial, the authors found that the precorneal retention time of 5 ocular lubricants ranged between 10 and 90 min, substantiating that the 2-h delay in the protocol could be relevant. A specific study to measure the residence time of the tested product would be of interest to confirm this hypothesis.

Some publications [11] highlight that SHs demonstrated antioxidant properties and ability to modulate inflammation. In an in vitro model, composed of human corneal cells, Fallacara et al.were able to measure a slight decrease in IL8-levels (pro-inflammatory marker) in SH-exposed tissues, compared to positive control. Moreover, Litwiniuk et al.reported a modulation of inflammatory activity due to a high molecular weight SH. On the contrary, a low molecular weight degradation product of SH would promote inflammation [12]. Besides, reduction in oxidative stress in the conjunctiva of human patients with dry eye disease has also been demonstrated with SH use [12]. It is presumed that high molecular weight SH absorbs reactive oxygen species thanks to its hydroxyl functional groups, or activates pathways related to the regulation of cellular redox status [12]. Though SH is not classified as an anti-inflammatory drug, we can wonder if the tested cross-linked component in our study could positively impact the secretions of lacrimal glands.

In our trial, we were not able to demonstrate an effect of the treatment on the quality of the tear film (TBUT score), which did not significantly differ over time. For tear supplements containing SH, several studies have demonstrated that the viscoelasticity of the polysaccharide leads to an increase in tear stability [2, 9]. We consequently expected TBUT to be improved at the end of the study.

Regarding viscoelasticity, the tested item is composed of cross-linked SH, a patented polymer showing higher consistency compared to the native form of SH [11]. In this original formulation, urea ensures the linkage between the different chains of SH, which increases its viscosity. Therefore, the contact time between SH and the ocular surface is extended and thus allows for less frequent applications [2]. Compared to other tear substitutes which require numerous daily instillations, the twice a day dosage is essential in increasing owner observance and ensuring therapeutic success [2].

Despite one occasional reaction (1 application) of redness in 2 dogs, we finally observed a good tolerance of the tested product throughout its use twice a day for 30 days., Besides the original cross-linked SH composition, the product is formulated without any preservative. A first in vitro study performed on human conjunctival cells has shown that quaternary ammonium compounds such as benzalkonium chlorides, benzododecinium bromide, or cetrimide were able to induce an apoptotic mechanism in cells at low concentrations, whereas a necrotic process was triggered at higher concentrations [13]. These harmful effects can be explained by the lipophilic nature of some preservatives (interfering with the membrane integrity of the corneal epithelium) as well as by the production of superoxide anions they induce. Overall, 3 mechanisms have been described: detergent effects causing loss of tear film stability, toxic effects to the corneal and conjunctival epithelia, and immune-allergic reactions [13]. Moreover, large retrospective studies carried out in thousands of human patients have revealed that subjective symptoms such as “discomfort upon instillation”, “burning-stinging”, “foreign body sensation”, “dry eye sensation”, “tearing” and “eyelid itching”, or clinical conjunctival, palpebral signs and superficial punctate keratitis, were significantly more frequent when using glaucoma medications with preservatives (*p* < 0.001) [14, 15]. Besides, in preserved medications, the occurrence of signs and symptoms was positively correlated with the number of instilled drops [14]. In animal species, the use of preserved anti-glaucoma eye drops on rabbits for 60 days has also shown to cause a greater reduction in TBUT and modifications in the corneal stroma (presence of oedema) compared to preservative-free anti-glaucoma eye drops [16]. This article also recalls that preservatives are linked to the onset of chronic fibrosis in the conjunctiva, or that they can cause microlesions of the corneal surface, as demonstrated in rabbits [16]. Thus, preservatives obviously contribute to the damage of the ocular surface and are susceptible to worsen clinical signs of keratitis. These considerations are of great importance, as tear substitutes must be administered for life.

## Conclusion

This pilot trial has demonstrated the benefits of a preservative-free cross-linked hyaluronic acid-based tear supplement, administered twice a day for one month, in dogs with dry eye. This product was able to significantly improve clinical signs, with a global ocular clinical score which significantly decreased within as little as two weeks of use (*p* < 0,0001). Moreover, the fact that it is preservative-free enhances the safety of the product. Together with the visible clinical improvement, an acceptable rhythm of product administration is essential to guaranteeing owner observance, hence therapeutic success. This will contribute to maintaining both the dog’s and the owner’s quality of life. These promising results would need to be further confirmed on a larger scale, in a double blinded controlled study and in a specific safety study with higher doses and/or frequencies of application.

## Data Availability

Data is available upon reasonable request to carole.gard@destaing.com.

## Abbreviations

KCS: Keratoconjunctivitis sicca
SH: Sodium hyaluronate
BID: Bis In Die: twice a day
TID: Ter In Die: three times a day
TBUT: Tear Break Up Time
STT1: Schirmer Tear Test 1
FT: Fluorescein Test
D: Day
QoL: Quality of Life
VICH: International Cooperation on Harmonization of Technical Requirements for Registration of Veterinary Medicinal Products
GCP: Good Clinical Practice
NSAIDs: Non-steroidal Anti-Inflammatory Drugs
SAIDs: Steroidal Anti-Inflammatory Drugs
LSD: Least Significant Difference
LOCF: Last Observation Carried Forward
